# Germline Variants in Patients Affected by Both Uveal and Cutaneous Melanoma

**DOI:** 10.1111/pcmr.13199

**Published:** 2024-09-24

**Authors:** Peter A. Johansson, Jane M. Palmer, Lindsay McGrath, Sunil Warrier, Hayley R. Hamilton, Timothy Beckman, Matthew G. D'Mellow, Kelly M. Brooks, William Glasson, Nicholas K. Hayward, Antonia L. Pritchard

**Affiliations:** ^1^ QIMR Berghofer Medical Research Institute Brisbane Queensland Australia; ^2^ University of Queensland Brisbane Queensland Australia; ^3^ Queensland Ocular Oncology Service The Terrace Eye Centre Brisbane Queensland Australia; ^4^ Department of Genetics and Immunology, Division of Biomedical Science University of the Highlands and Islands Inverness Scotland UK

**Keywords:** cutaneous melanoma, genetic risk, germline variants, sequencing, uveal melanoma

## Abstract

Uveal melanoma (UM) and nonacral cutaneous melanoma (CM) are distinct entities with varied genetic landscapes despite both arising from melanocytes. There are, however, similarities in that they most frequently affect people of European ancestry, and high penetrance germline variants in *BAP1*, *POT1* and *CDKN2A* have been shown to predispose to both UM and CM. This study aims to further explore germline variants in patients affected by both UM and CM, shedding light on the underlying genetic mechanism causing these diseases. Using exome sequencing we analysed germline DNA samples from a cohort of 83 Australian patients diagnosed with both UM and CM. Eight (10%) patients were identified that carried pathogenic mutations in known melanoma predisposition genes *POT1*, *MITF*, *OCA2*, *SLC45A2* and *TYR*. Three (4%) patients carried pathogenic variants in genes previously linked with other cancer syndromes (*ATR*, *BRIP1* and *MSH6*) and another three cases carried monoallelic pathogenic variants in recessive cancer genes (xeroderma pigmentosum and Fanconi anaemia), indicating that reduced penetrance of phenotype in these individuals may contribute to the development of both UM and CM. These findings highlight the need for further studies characterising the role of these genes in melanoma susceptibility.

## Graphical abstract

Germline variants predisposing uveal and cutaneous melanoma are identified using exome sequencing. Created with BioRender.com.

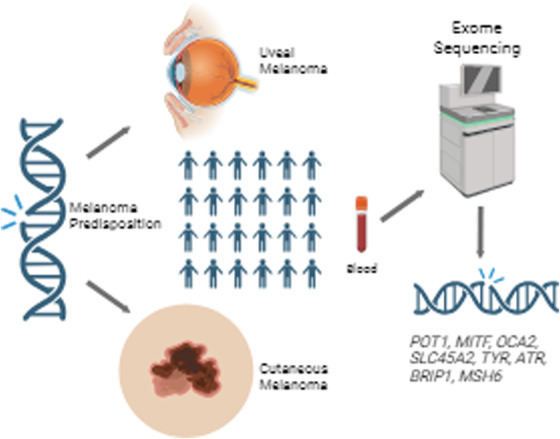

**Summary**

- This study explored the genetic variants in individuals affected by both uveal and cutaneous melanoma.
- It confirmed that both subtypes are part of the *POT1* tumour predisposition syndrome spectrum.
- Variants in established cutaneous melanoma genes were observed, suggesting they could also predispose to uveal melanoma.
- Some patients carried variants in genes previously linked to other cancers, potentially expanding the disease spectra pathogenic variants in these genes predispose to.

Uveal melanoma (UM) originates from melanocytes in the uveal tract of the eye and differs from cutaneous melanoma (CM) in anatomical location, molecular drivers and clinical characteristics. CM arise from the melanocytes in the layer of basal cells at the deepest part of the epidermis of the skin and when occurring in nonglabrous skin are frequently driven by exposure to ultraviolet (UV) radiation. While immunotherapy has radically changed the outcome of advanced‐stage CM, its success has been more limited in UM (Nathan, Hassel, et al. 2021). This discrepancy can be partly attributed to the considerably lower tumour mutation burden in UM (Newell et al. 2022; Rizvi et al. 2015), but differences in the tumour microenvironment might also contribute. CM often exhibit hotspot mutations in *NRAS* or *BRAF*, which constitutively activate the mitogen‐activated protein kinase (MAPK) pathway. In contrast, UM frequently harbours mutations in *GNAQ* or *GNA11*, which activate the G‐protein signalling pathway. These differences underscore the distinct molecular pathways driving the development of UM and CM.

Despite these differences, there are also some similarities, particularly in factors influencing predisposition. Epidemiological studies have shown that UM and (nonacral) CM most frequently affect people of European ancestry. In CM, this association is caused by fair skin, offering less protection against mutagenic UV radiation (Del Bino, Sok, and Bernerd 2013), supported by genome‐wide association studies of CM, which have identified numerous susceptibility loci near pigmentation genes (Landi et al. 2020). Similarly, smaller association studies have identified four loci associated with UM (Ferguson et al. 2016; Mobuchon et al. 2017, 2022; Thomsen et al. 2020), with three overlapping CM susceptibility regions, including the loci at *IRF4* and *HERC2*/*OCA2* linked with pigmentation and the *TERT*/*CLPTM1L* locus associated with telomere regulation (Derrien et al. 2022). This suggests an overlap in the molecular mechanisms underlying CM and UM tumorigenesis, which is further supported by shared rare high penetrance variants predisposing to both tumour types in families. Protein‐truncating variants (PTVs) in *BAP1* dramatically increase the risk for both UM and CM (Abdel‐Rahman et al. 2011), as part of the BAP1 tumour predisposition syndrome (Walpole et al. 2018) and variants in *POT1* and *CDKN2A* predispose to CM (Robles‐Espinoza et al. 2014), while case reports have suggested that *POT1* and *CDKN2A* are risk genes for UM (Hearle et al. 2003; Kannengiesser et al. 2003; Nathan et al. 2021), although with limited penetrance (Abdel‐Rahman et al. 2020).

Given these shared aetiologies, we hypothesised that other yet to be identified germline variants predispose to both UM and CM. To explore this, we recruited 83 patients attending a single Australian ocular oncology referral centre diagnosed with both UM and CM and performed genome or exome sequencing.

This study was approved by the Human Research Ethics Committee of the QIMR Berghofer Medical Research Institute (HREC/14/QPAH/495). Blood or saliva samples were collected with informed written consent. Blood DNA was extracted using standard salting‐out methods, and saliva DNA was extracted using Oragene DNA kits (OG‐500; DNA Genotek, Ottawa, Canada) according to the manufacturer's instructions. Genome and exome (using SureSelect V7 postcapture kits) sequencing was conducted by Macrogen (Seoul, South Korea) on the Illumina platform. The resulting sequence reads were aligned to the HumanG1Kv37 assembly using the Burrows‐Wheeler Aligner (BWA‐MEM) (Li and Durbin 2010) and duplicate reads were identified by Picard v1.141 (https://broadinstitute.github.io/picard/). To enhance the alignments, reads were realigned against common insertions and deletions and base qualities were calibrated using the Genome Analysis Toolkit (GATK) (McKenna et al. 2010). Small substitutions were detected with bcftools (Li and Durbin 2009), while indels were detected using Pindel (Ye et al. 2009), and annotated using the Ensembl Variant Effect Predictor (VEP) (McLaren et al. 2016) including Sorting Intolerant From Tolerant (SIFT) and Polymorphism Phenotyping v2 (PolyPhen2) predictions (Adzhubei, Jordan, and Sunyaev 2013; Ng and Henikoff 2003) and variant allele frequencies (VAFs) from the Genome Aggregate Database (gnomAD) (Karczewski et al. 2020), and variants were subsequently annotated with Combined Annotation Dependent Depletion (CADD) (Kircher et al. 2014), Rare Exome Variant Ensemble Learner (REVEL) (Ioannidis et al. 2016) and MetaRNN (Li et al. 2022) predictions using dbNSFP v4.8 (Liu et al. 2020), with CI‐SpliceAI predictions (Strauch et al. 2022), and with ClinVar data (downloaded 2 May 2024). High‐quality variants were identified by requiring at least three variant reads and phred‐scaled likelihood for wildtype > 70. We removed variants that were located in segmentally duplicated regions (Bailey et al. 2002), showed strand bias (*p* < 1 × 10^−4^), tail distance bias (*p* < 1 × 10^−4^), clustered near the end of the reads (< 10%), or if the variant reads had substantially lower average mapping quality than the wild‐type reads (> 30). Nonsynonymous *MC1R* variants were categorised into R alleles, strongly associated with red hair, and r alleles weakly associated with red hair, as described elsewhere (Robles‐Espinoza et al. 2016; Valverde et al. 1995).

Of the 83 patients, 42 (51%) were male. The median age at diagnosis of the first CM was 64.9 years (range 22.4–89.1) and for UM was 67.7 years (range 27.3–90.1); (Table 1; Table S1). One patient with a particularly notable history of bilateral UM (56 and 73 years) and three CMs had no coding region mutation that could be linked with a heightened melanoma risk.

**TABLE 1:** Patient characteristics.

Sex, male (%)	42 (51)
Number of CM, mean (range)	1.57 (1–7)
Onset age 1st CM, median (range)	64.9 (22.4–89.1)
CM Histotype, *n* (%)	
Malignant melanoma, NOS	29 (22)
Melanoma in situ	31 (24)
Nodular melanoma	7 (5.4)
Superficial spreading melanoma	30 (23)
Superficial spreading melanoma in situ	30 (23)
CM Primary site, *n* (%)	
Face	6 (4.7)
Lower limb (including hip)	25 (20)
Scalp and neck	12 (9.4)
Trunk	44 (35)
Upper limb (including shoulder)	40 (31)
Number of UM, mean (range)	1.01 (1–2)
Onset age 1st UM, median (range)	67.7 (27.3–90.1)
UM Primary site, *n* (%)	
Choroid	63 (75)
Ciliary body	21 (25)
*MC1R*, *n* (%)	
Wildtype	15 (18)
r/0	16 (19)
r/r	8 (9.6)
R/0	22 (27)
R/r	14 (17)
R/R	8 (9.6)
Relatives with cancer, mean (range)	1.4 (0–12)

*Note:* Characteristics of the patient cohort including number of, histotype and location of cutaneous melanoma (CM) and uveal melanoma (UM). R alleles of *MC1R* are strongly associated with red hair colour, while r alleles and wildtype (0) are not. Near relatives include first and second‐degree relatives with confirmed diagnosis of cancer. See also Table S1.

Next, we investigated variants in established melanoma predisposition genes. Two patients carried variants in *POT1* (c.1851_1852del p.(D617Efs*9), c.670G>A p.(D224N)). The p.(D224N) variant was classified in ClinVar as variant of uncertain significance (VUS), but notably it has previously been observed in familial melanoma and patients with multiple primary melanomas (Shi et al. 2014). The p.(D617Efs*9) variant had conflicting classifications in ClinVar as submitters had classified it as VUS and likely pathogenic. Both variants have been described in several other cancer patients, including those with glioma, colorectal and lung cancer (reviewed in Nathan et al. 2021), and we therefore consider them likely to result in a loss of POT1, which contributes to the melanoma risk. Six patients carried pathogenic variants in genes known to be associated with CM, including *MITF* (c.952G>A p.(E318K)), *OCA2* (c.1327G>A p.(V443I)), *SLC45A2* (c.606G>C p.(W202C), c.834C>G p.(T278*)) and *TYR* (c.626C>T p.(P209L), c.1118C>A p.(T373K)) (Bertolotto et al. 2011; Nathan et al. 2019; Yokoyama et al. 2011) (Table 2, Table S2). Another 17 variants, classified as conflicting or VUS in ClinVar, were observed in genes associated with CM: *ATM*, *BAP1, MITF*, *OCA2*, *POT1*, *SLC45A2* and *TERT* (Table S2). These genes are mostly involved in pigmentation; therefore, variants could affect the level of protection against UV radiation. A gene strongly associated with pigmentation is *MC1R*. Patients with a history of CM and UM had on average 0.63 *MC1R* R alleles, almost identical to a cohort of CM patients (0.64), and more frequent than in a UM cohort (0.48) (Newell et al. 2022). The observation that R alleles are more common in CM than UM (Fisher's exact, *p =* 0.03) is consistent with R alleles (associated with red hair, pale skin and poor tanning ability) not being associated with UM risk but strongly associated with the development of CM (Landi et al. 2020).

**TABLE 2:** Selected variants.

Sample ID	Gene	Amino acid change^e^	ClinVar classification^f^	GnomAD VAF^g^	Recessive disorder
MELA_0959	ATR^a^	p.(I774Nfs*3)	PV or LPV	4.7 × 10^−5^	
MELA_0927	BRIP1^b^	p.(R798*)	PV or LPV	2.0 × 10^−4^	
MELA_0940	MITF^a^	p.(E318K)	PV or LPV; RF	2.4 × 10^−3^	
MELA_0927	MSH6^b^	p.(R1076C)	LPV	3.1 × 10^−5^	
MELA_0886	MSH6^b^	p.(R1076C)	LPV	3.1 × 10^−5^	
MELA_0919	POT1^c^	p.(D617Efs*9)	CC	3.1 × 10^−5^	
MELA_0921	POT1^c^	p.(D224N)	VUS	2.0 × 10^−4^	
MELA_0928	ERCC5^b^	p.(K917Nfs*65)	PV	0	XP^h^ group G
MELA_0941	FANCE^b^	p.(R371W)	PV or LPV	6.2 × 10^−5^	Fanconi anaemia
MELA_0951	OCA2^a^	p.(V443I)	PV or LPV	6.1 × 10^−3^	Oculocutaneous albinism type 2
MELA_0907	SLC45A2^a^	p.(Y278*)	PV or LPV	1.0 × 10^−4^	Oculocutaneous albinism type 4
MELA_0926	SLC45A2^a^	p.(W202C)	PV or LPV	1.0 × 10^−4^	Oculocutaneous albinism type 4
MELA_0936	TYR^a^	p.(P209L)	PV	1.5 × 10^−5^	Oculocutaneous albinism types 1, 3
MELA_0927	TYR^a^	p.(T373K)	PV	1.0 × 10^−3^	Oculocutaneous albinism types 1, 3
MELA_0888	WRN^d^	p.(D77H)	VUS	5.0 × 10^−4^	Werner syndrome
MELA_0950	WRN^d^	p.(R711W)	CC	3.0 × 10^−4^	Werner syndrome
MELA_0944	WRN^d^	p.(H916P)	VUS	2.0 × 10^−4^	Werner syndrome
MELA_0955	WRN^d^	p.(I1145T)	VUS	0	Werner syndrome
MELA_0910	XPA^b^	p.(M1?)	LPV	0	XP^h^ group A

*Note:* See also Table S2 for more details.

Abbreviations: CC, conflicting classifications; LPV, likely pathogenic variant; PV, pathogenic variant; RF, risk factor; VUS, variant of uncertain significance.

^a^ Gene associated with cutaneous melanoma.

^b^ Gene associated with various cancers.

^c^ Gene linked with cutaneous and uveal melanoma.

^d^ Gene linked with acral melanoma.

^e^ The variant's predicted change in the protein encoded by the MANE select transcript except for MITF for which the change is displayed in the protein mainly expressed in melanoma, NP_000239.1.

^f^ The aggregate classification in the ClinVar database as of 2^nd^ May 2024.

^g^ The variant allele frequency in the non‐Finnish European population in version 3 of the Genome Aggregation Database.

^h^ Xeroderma pigmentosum.

Four patients carried rare (gnomAD VAF ≤ 5 × 10^−4^) missense variants in *WRN* (c.229G>C p.(D77H), c.2131C>T p.(R711W), c.2747A>C p.(H916P), c.3434T>C p.(I1145T)), a gene associated with Werner syndrome and acral melanoma (Goto et al. 1996; Newell et al. 2022). The majority of in silico predictions for p.(D77H) and p.(I1145T) were benign, 3/5 were pathogenic for p.(R711W), and 4/5 were pathogenic for p.(H916P), consistent with that the p.(H916P) variant affects a highly conserved region within the functionally crucial zinc‐binding subdomain (Kitano 2014).

Next, we investigated genes with common somatic alterations in melanoma. Three patients carried rare missense variants in *GNAQ* (c.93G>C p.(R31S)) or its paralogue *GNA11* (c.323C>G p.(A108G), c.434A>T p.(Y145F)), two genes typically associated with driving the development of UM via mutations at hotspots p.R183 and p.Q209 (Robertson et al. 2017; Van Raamsdonk et al. 2010). Most in silico predictions were benign for these variants (Table S2) except that *GNA11* p.(Y145F) was predicted pathogenic by 3/5 tools, which suggests it possibly alters the protein function and contributes to the melanoma risk.

Three patients carried four variants in *SPRED1*, which encodes a tumour suppressor regulating the MAPK pathway and is often disrupted through somatic mutations in mucosal melanoma (Ablain et al. 2018). One individual carried c.875A>G p.(Y292C) and c.887G>A p.(C296Y) variants on the same chromosome, in line with gnomAD data in which these two variants always co‐occur (*n* = 18/2,82,666 alleles). These two variants were classified as ‘Conflicting Classifications’ in ClinVar, all in silico methods predicted the p.C296Y variant to be benign, while the p.Y292C variant was predicted to be pathogenic by 4/5 tools, which suggests the haplotype might affect the function of SPRED1.

We next looked at genes associated with other cancers (Table S3) and focused on rare (gnomAD VAF ≤ 5 × 10^−4^) pathogenic variants. Six patients carried at least one pathogenic variant in cancer‐related genes. This included two patients carrying PTVs in the DNA repair genes *ATR* or *BRIP1*. PTVs in *ATR* have been associated with oropharyngeal cancer and nonmelanoma skin cancer (Tanaka et al. 2012); the carrier in our cohort had bilateral breast cancer (62 and 68 years) in addition to UM and CM. PTVs in *BRIP1* have been associated with ovarian cancer (Rafnar et al. 2011); the carrier in our cohort had no additional primary cancer diagnoses (49 years). Two patients carried the likely pathogenic variant c.3226C>T p.(R1076C) in *MSH6*, which has been reported to predispose to Lynch syndrome. Pathogenic variants in *MSH6* have been associated with cancers other than colorectal carcinomas, including endometrial (Hendriks et al. 2004) and nonmelanoma skin cancers (Barrow et al. 2009); however, melanomas have yet to be confirmed in the tumour predisposition spectrum (Liu et al. 2022). One carrier of the p.(R1076C) variant had developed lymphoma (76 years), while the other had not developed any additional primary cancers (49 years). Other pathogenic variants were present in *FANCE*, *XPA* and *ERCC5*, associated with Fanconi anaemia, xeroderma pigmentosum (XP) group A and XP group G, respectively. Patients with Fanconi anaemia can have skin pigmentation and ophthalmic anomalies, as well as increased risk of cancers, including haematological malignancies and skin cancers (Mehta and Ebens 1993). Aside from UM and CM the carrier in our cohort had not developed any further malignancies by their seventh decade. XP is a recessive disorder leading to > 10,000 times greater risk of skin cancers, > 2000 times greater risk of melanoma (Bradford et al. 2011) and patients frequently experience ocular anomalies due to UV radiation damage (Kraemer, Lee, and Scotto 1987). While XP is a recessive disorder caused by having pathogenic variants in both copies of the gene, it is plausible that carrying a single pathogenic variant is associated with a milder phenotype such as an increased risk of melanoma. There is a precedent for this, with variants causing the recessive oculocutaneous albinism syndromes increasing CM risk in carriers of one pathogenic allele in melanoma families (Goldstein et al. 2017; Nathan et al. 2019).

One patient carried pathogenic variants in *FBXW7* (c.1393C>T p.(R465C)) and *KRAS* (c.437C>T p.(A146V)), which have frequently been observed as somatic mutations in haematological malignancies (COSMIC IDs: COSV55891008 and COSV55498939). This patient was diagnosed with B cell chronic lymphocytic leukaemia 20 years prior to sequencing, which means these variants may have arisen somatically.

In conclusion, eight (10%) UM/CM patients carried a pathogenic mutation in high or medium‐penetrance melanoma predisposition genes and three (4%) cases carried variants known to predispose to other cancers. Another three cases carried monoallelic variants in cancer genes associated with recessive disorders (XP and Fanconi anaemia), indicating that reduced penetrance of phenotype in these individuals may increase their risk of both UM and CM. While limited by the cohort size, this investigation highlights the need for additional studies to examine the role of this group of genes in melanoma predisposition.

## Author Contributions

Conceptualisation and supervision: N.K.H.; data curation: P.A.J., J.M.P., H.R.H., A.L.P.; formal analysis and software: P.A.J.; funding acquisition: J.M.P., W.G., N.K.H.; methodology and writing – original draft preparation: P.A.J., A.L.P.; resources: L.M., S.W., T.B., W.G.; investigation: M.G.D., K.M.B.; writing – review and editing: P.A.J., J.M.P., L.M., S.W., H.R.H., T.B., M.G.D., K.M.B., W.G., N.K.H. and A.L.P.

## Conflicts of Interest

The authors declare no conflicts of interest.

## Supporting information


**Table S1.** Patient characteristics.

**Table S2.** Rare nonsynonymous germline variants in cancer‐associated genes.

**Table S3.** List of cancer‐associated genes analysed.

## Data Availability

Sequence data has been deposited at the European Genome‐phenome Archive and are available under accession numbers EGAS00001001552 and EGAS00001007584.
